# Is Conservative Surgery the Best Approach for Peripheral Calcifying Epithelial Odontogenic Tumors?

**DOI:** 10.2174/1874210601812010856

**Published:** 2018-10-25

**Authors:** Isadora Luana Flores, Tissiana Rachel Rossi Schneider, Ana Carolina Uchoa Vasconcelos, Sandra Beatriz Chaves Tarquinio, Ricardo Alves de Mesquita, Ana Paula Neutzling Gomes

**Affiliations:** 1 Department of Conservative Dentistry, Oral Pathology, Federal University of Rio Grande do Sul, Rua Ramiro Barcelos 2492, Santa Cecília, Porto Alegre, RS, Brazil; 2 Pelotas Dental School, Semiology and Clinic, Federal University of Pelotas, Rua Gonçalves Chaves, 457, Bairro Centro, Pelotas, RS, Brazil; 3 Department of Oral Pathology and Surgery, Federal University of Minas Gerais, Belo Horizonte, Rua Prof Moacir Gomes de Freitas, 688, Bairro Pampulha, Belo Horizonte, MG, Brazil

**Keywords:** Calcifying epithelial odontogenic tumor, Clear cell, Peripheral, Oral diagnosis, Surgery, Treatment

## Abstract

**Background:**

Peripheral Calcifying Epithelial Odontogenic Tumors (CEOT) rich in clear cells are a rare entity in the oral cavity, with only 14 previous case reports in the English literature. None have discussed recommended treatment approaches for extraosseous CEOT.

**Objective:**

This brief descriptive review describes a treatment approach for peripheral CEOT including the clear cell variant.

**Study design:**

A complete review of all well-documented extraosseous case reports with an emphasis on the treatment was performed. Additionally, the present article reports a case of a 21-year-old woman with an asymptomatic swelling in the gingiva finally diagnosed as peripheral CEOT abundant in clear cells.

**Results:**

Twenty-four cases of peripheral CEOT were described; conservative surgery was the first treatment approach in approximately 80% of cases, with only one recurrence.

**Discussion:**

Clear cell finding was not associated with more aggressive behavior.

**Conclusion:**

Conservative surgery may be an advantageous approach for this group of peripheral lesions with or without clear cells, with a recurrence rate of approximately 4%.

## 1 INTRODUCTION

Calcifying Epithelial Odontogenic Tumor (CEOT) is an uncommon jaw lesion with a benign and slow-growing pattern but locally aggressive course [1]. The lesions are most prevalent among patients aged 30 to 50 years, and no sex predilection has been observed [1, 2]. Clinically, the CEOT appears as a slowly asymptomatic expansion with radiolucent honeycomb appearance in the posterior areas of the mandible [2]. The CEOT is predominantly an intraosseous tumor in approximately 94% of cases [3]. The peripheral variant is a rare tumor (6%) with less aggressive behavior that was described by Pindborg in 1966 [4]. The classical histopathological aspects include sheets and islands of eosinophilic polyhedral epithelial cells in association with homogeneous pink amyloid-like deposits and areas of calcification [5-22]. One unusual histological finding is the presence of clear cells, as reported in 14 cases of peripheral CEOT in the English literature [2, 5-19, 22, 23].

CEOT is an odontogenic tumor; the consensus regarding the best therapeutic approach is surgical excision with safe margins recommended for the intraosseous variant [3, 4]. However, the peripheral lesions are commonly treated through conservative surgery with no review of this approach [3]. The present study reports a case of a 21-year-old Caucasian female patient with a mandibular peripheral CEOT rich in clear cells. An additional critical literature review focuses on the treatment protocols for the peripheral variant of the tumor.

## 2 CASE DESCRIPTION AND RESULTS

**Fig. (1A and B):**
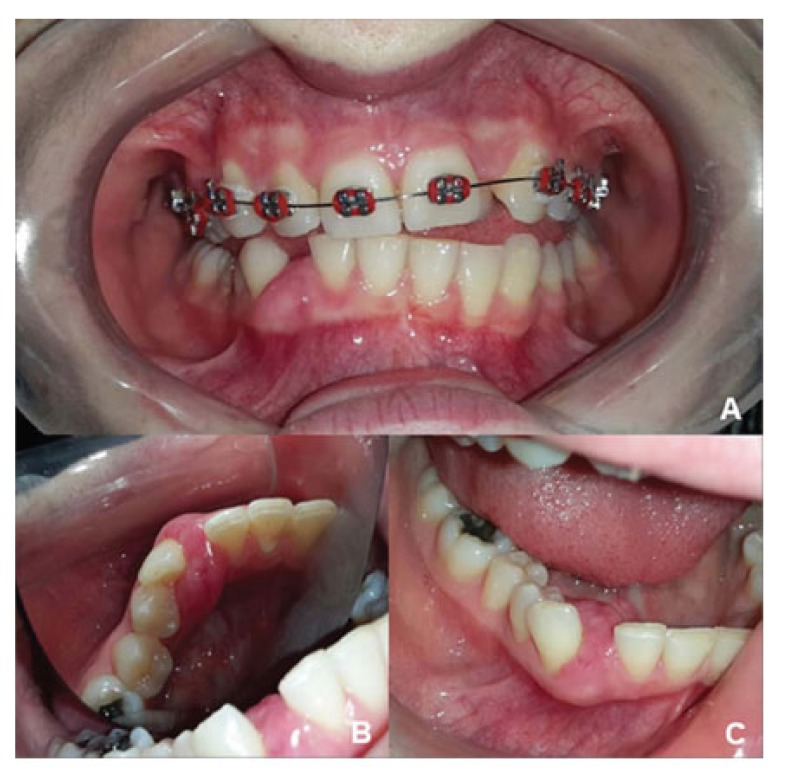
Well-defined nodule with focal erythematous area in vestibular and lingual gingiva between the mandibular right lateral incisor and the right canine.

A 21-years-old Caucasian woman presented to a private dental clinic with a chief complaint of asymptomatic swelling in the gingiva observed four years prior. A gradual increase in size and no history of previous treatment were also reported during the anamnesis. The patient signed the informed consent, which represents the ethical approval of the faculty committee. Her medical and socio-economic histories were not contributory. The extra-oral evaluation did not reveal changes. The intraoral examination revealed a sessile nodule with a color similar to that of the mucosa and a focal erythematous area with a fibro-elastic consistency measuring 1.5 cm in the largest diameter extending from the inferior right lateral incisor to the inferior right first premolar. The lesion involved the vestibular and lingual gingiva, causing displacement of the inferior right canine (Fig. **1**).

**Fig. (2):**
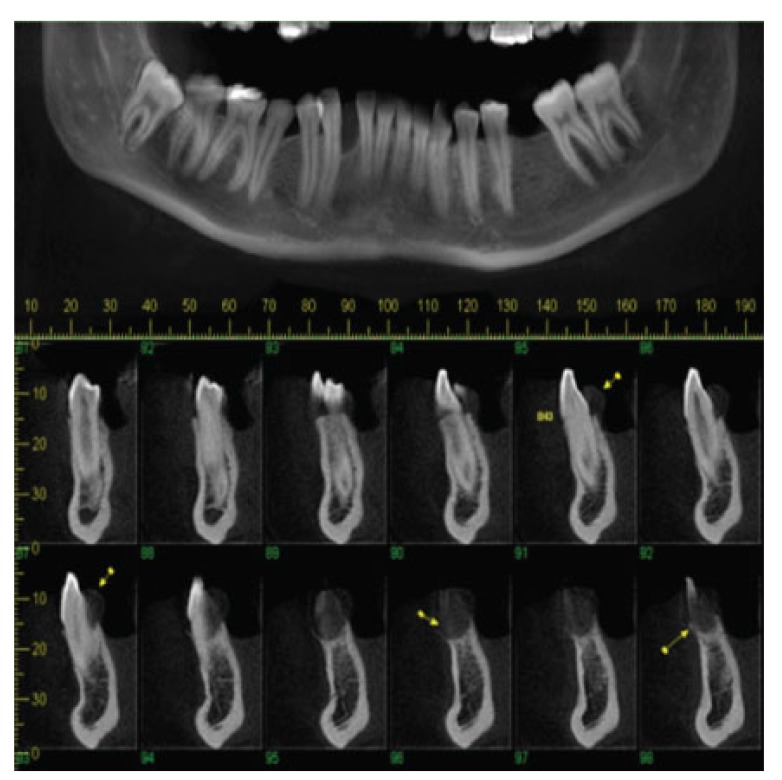
The panoramic reconstruction of Cone Beam Computed Tomography (CBCT) showed a radiolucent area between the inferior right lateral incisor and the inferior right canine. Note the displacement of the 43 tooth. Parasagittal slices of CBCT confirmed the soft tissue origin with clear overlap of lesion on the alveolar bone. The yellow arrow in 85 and 87 slices indicating the peripheral CEOT. Reabsorption of the alveolar crest is observed in 88 to 92 and highlighted on 90 and 92 slices (yellow arrow).

**Fig. (3):**
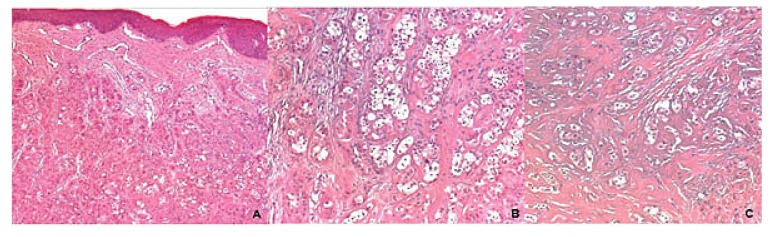
**A –** Low power magnification showed nests and cords of odontogenic epithelial cells in the dense fibrous connective tissue under the mucosal epithelium (H&E, 50X original magnification). **B.** Varied size of epithelial islands composed by polyhedral cells with the presence of clear cells are also observed (H&E, 400X original magnification). **C.** Note the marked eosinophilic deposit interspersing the clear cell component (H&E, 400X original magnification).

**Fig. (4):**
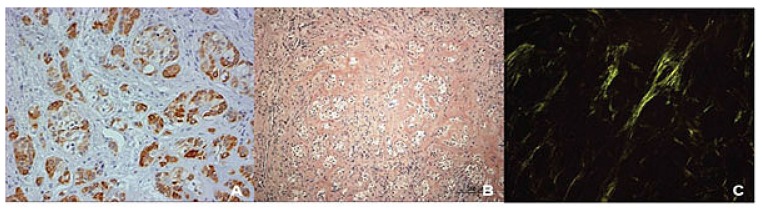
**A –** Immunohistochemistry for CK19 showed positive labelling for odontogenic epithelium except to clear cells component (400X original magnification). Congo-Red positivity **(B**- 50X original magnification) and apple-green birefringence under the polarized light for amyloid-like deposits (**C-** 50X original magnification).

**Fig. (5):**
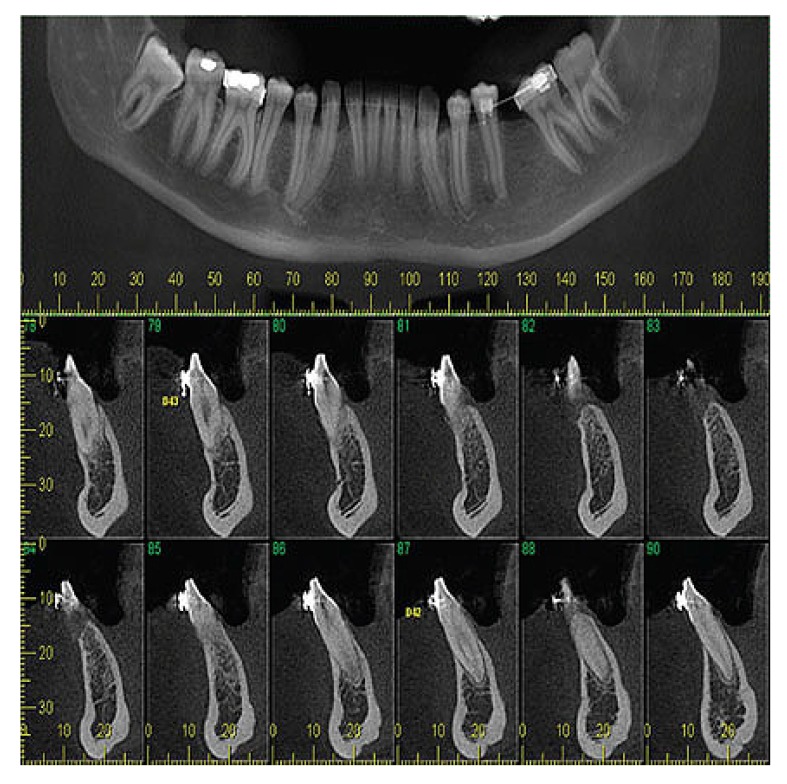
Panoramic reconstruction and parasagittal slices of CBCT showed no signs of lesion and bone neoformation is reached after 15 months of conservative approach.

Panoramic reconstruction and parasagittal slices of the Cone Beam Computed Tomography (CBCT) showed a slightly superficial hypodense area between the inferior right lateral incisor and inferior right canine with reabsorption of the alveolar crest (Fig. **2**). Based on the clinical and immunological aspects, the main diagnosis hypotheses included peripheral ossifying fibroma, peripheral giant cell lesion, and ancient pyogenic granuloma. The peripheral odontogenic tumors were also included as a differential diagnosis. An excisional biopsy was performed and a clear separation was noted between the lesion and mandible bone during the trans-surgical approach. The histopathological analysis revealed a well-circumscribed proliferation comprising numerous islands and strands of epithelial polyhedral cells with well-defined borders and marked round nucleus in the connective tissue under the mucosal epithelium. Numerous nests, cords, and small islands of polyhedral cells with clear and vacuolated abundant cytoplasm were observed interspersed with the amorphous eosinophilic deposits (Fig. **3**). Immunohistochemistry was performed, which yielded positive results for CK-19 in the epithelial cells, except for the clear cells. Congo red staining showed the presence of amyloid-like deposits with apple-green birefringence under polarized light (Fig. **4**). A final diagnosis of a peripheral CEOT rich in clear cells was reached. No complications were observed in the postoperative appointment and a follow-up schedule was established. The patient has had no recurrence after 22 months (Fig. **5**).

## 3 PERIPHERAL CEOT CASES

Long-term follow-up of the central variant of CEOT has shown the best results, with no recurrence following block resection with safe margins; however, well-documented cases are scarce [1, 24]. Additionally, there is discussion regarding aggressive behavior involving clear cells in the CEOT [1, 21, 24]. Other odontogenic lesions with clear cells including ameloblastoma and clear cell odontogenic tumors have been reclassified as malignant, in which a more aggressive course can be expected [24, 25]. Nonetheless, this premise has not been confirmed for CEOT because the latest review of all central variants showed no conclusive data regarding the worse course for this type of lesion [1, 24, 25].

Peripheral CEOT is considered a harmless lesion, but the recommended approach for peripheral cases in the presence or lack of clear cells is not emphasized in previous reports [2, 4-6, 8-20, 22]. In this context, a search of the English literature was performed in the PubMed database using the keywords “calcifying epithelial odontogenic tumor” with “Pindborg,” “peripheral,” “clear cell,” and “treatment.” All manuscripts on peripheral CEOT published until May 2018 were considered. Cross-references were included. Studies involving mixed odontogenic tumors in association with CEOT, CEOT associated with other conditions, no exclusive extraosseous tumors, absence of treatment modality, and no full-text database were excluded. The anatomic sites, duration, clinical and imaging aspects, types of treatment, recurrence, and follow-up are summarized in Table **1**.

## 4 DISCUSSION

The peripheral variant is a rare presentation of CEOT, with a differential diagnosis including gingival reactive lesions such as ancient pyogenic granuloma, peripheral giant cell lesion, and peripheral ossifying fibroma in addition to other peripheral odontogenic tumors. The histopathological aspects of the extraosseous are similar to those of the intraosseous CEOT counterparts and Congo red staining confirms the presence of amyloid-like material such as immunohistochemistry keratin markers confirm the odontogenic epithelial origin [21, 22]. In addition, clear cells have been observed in some odontogenic lesions and, although previous authors have speculated their relationship with more aggressive CEOT, no role has yet been shown for the relationship between the behavior and this histopathological presentation [21, 22]. Extraosseous CEOT is considered a less aggressive tumor and conservative surgery is performed in most cases. However, no previous articles have evaluated the best approach for peripheral CEOT based on recurrence and follow-up data. The current review identified no clinical significance in relation to the aggressive aspects. This finding is supported by the observation that approximately 80% of cases were treated with conservative management, with only one recurrent case without a clear cell component [2, 4-6, 8-18, 22]. A detailed evaluation of all articles on peripheral CEOT found no reported recurrence during follow-up in 96% of cases [2, 5, 6, 8-20, 22, 23, 26].

A conservative soft tissue excision was the main approach in the literature and was also sufficient for the complete resolution of the current case. These findings suggest an indolent, local, non-infiltrative course of peripheral lesions when compared to intraosseous CEOT. The discrete cupping or erosion of superficial bone may be caused by compression rather than by invasive behavior, supporting the non-aggressive behavior of the lesion. Other peripheral odontogenic tumors, including ameloblastoma, ameloblastic fibroma, calcifying cystic odontogenic tumor, and adenomatoid odontogenic tumor, present similar characteristics [27, 28]. An isolated case with recurrence was observed with posterior intraosseous involvement; however, it was a unique case with bilateral peripheral lesions [18]. Clear cells were also not observed and there was no recurrence after the second conservative surgery [18].

## CONCLUSION

Our review confirms that conservative enucleation is the most appropriate management for peripheral CEOT. Moreover, we identified no association between clear cells and clinical aggressiveness in peripheral lesions as was previously suggested for the central counterparty.

## Figures and Tables

**Table 1:** Previous articles about peripheral calcifying epithelial odontogenic tumor including clear cell variant with emphasis in the treatment.

**Authors**	**Anatomic Site**	**Duration**	**Clinical Presentation**	**Imaging** **Presentation**	**Treatment**	**Recurrence**	**Folow-up**
Pindborg, 1966^5^	Max. gingiva	5 years	Painless firm mass	NCI	Simple excision	No	NCI
Abrams and Howell, 1967^10^**	Mand. gingiva	NA	Painless firm mass	Crest resorption	Simple excision	No	3 years
Decker and Laffitte, 1967^11^	Mand. gingiva	5years	Painless firm mass	NCI	Simple excision	No	NCI
Patterson *et al*, 1969^12^	Mand. gingiva	1 year	Painless firm mass	NCI	Simple excision	No	NCI
Krolls and Pindborg, 1974^13^	Mand. gingiva	NA	Painless firm mass	NCI	Simple excision	No	NCI
Wherteimer *et al*., 1977^14^**	Max. gingiva	NA	Painless firm mass	No	Simple excision	No	NA
Ai-ru *et al*., 1982^15^**	Mand. gingiva	10 years	Painless firm mass	NCI	Ressection	No	2 years
Ai-ru *et al*., 1982^15^**	Mand. gingiva	2 years	Painless firm mass	No	Partial ressection	No	10 years
Takeda *et al*., 1983^16^	Max gingiva	NA	Painless firm mass	NCI	Excision underlying bone	No	NCI
KH Ng *et al*., 1996^17^	Max. gingiva	1 year	Painless firm swelling	Erosion	Excision	No	NA
Houston & Fowler, 1997^18^**	Max. gingiva	5 months	Ulcerated mass	No	Simple excision	No	4 years
Houston & Fowler, 1997^18^**	Mand. gingiva	5 months	Ulcerated mass	Erosion*	Simple excision	No	4 years
Orsini *et al*., 2000^9^**	Max. gingiva	6 months	Painless red mass	Not performed	Simple excision	No	4 years
Mesquita *et al*., 2003^6^**	Max. gingiva	10 months	Painless firm nodule	No	Excision	No	2.5 years
de Oliveira *et al*., 2009^2^**	Max. gingiva	NA	Painless exophitic mass	No	Excision	No	1 year
de Oliveira *et al*., 2009^2^**	Mand. gingiva	NA	Painless exophitic mass	Superficial cupping	Excision	No	1 year
Abrahão *et al*., 2009^19b^	Mand gingiva	3 months	Painfull erithematous swelling	No^a^	Simple excision^c^	Yes^d^	3.5 years
Habibi *et al*., 2009^20^**	Max. gingiva	11 years	Ulcerated mass	NCI	Excisional biopsy with 5-mm safety margins	No	NA
Marino *et al*., 2013^21^	Max. gingiva	NA	Painless swelling	Bone resorption	Conservative surgery including teeth extraction	No	2 years
Afrogheh *et al*., 2014^7^**	Mand. gingiva	6 months	Painless swelling	Erosion	Complete excision	No	1.5 years
Shetty *et al*., 2016^22^**	Mand. gingiva	8 months	Painless swelling	No	Excisional biopsy	No	6 months
de Carvalho *et al*., 2016^26^	Mand. gingiva	1 month	Painless swelling	No	Excisional biopsy	No	1 year
Bajpai M, 2018^23^**	Max. gingiva	9 months	Pink-coloured swelling	Loss of lamina dura	Complete excision	No	6 months
Flores *et al*., 2018**¡	Mand. gingiva	4 years	Painless swelling	Superficial hypodense cupping	Conservative surgery	No	22 months

Max.= maxillary Mand. = mandible NA = Not available NCI = Not clearly identified a = osseous resorption and calcifications (recurrent lesions) b = bilateral c = soft tissue excision followed bone curettage d = after 1 year *Observed only during the surgical procedure **Clear cell variant ¡ Current case
